# A rapid method for measuring serum oxidized albumin in a rat model of proteinuria and hypertension

**DOI:** 10.1038/s41598-019-45134-x

**Published:** 2019-06-13

**Authors:** Beibei Liu, Keiko Yasukawa, Suang Suang Koid, Alimila Yeerbolati, Latapati Reheman, Conghui Wang, Yutaka Yatomi, Tatsuo Shimosawa

**Affiliations:** 1 Department of Clinical Laboratory, The University of Tokyo Hospital, Tokyo, Japan; 2 Department of Clinical Laboratory, International University of Health and Welfare School of Medicine, Chiba, Japan

**Keywords:** Animal disease models, Experimental models of disease

## Abstract

Oxidative stress is a risk for and cause of various disease, however, measurements of oxidative stress are either time-consuming or non-specific. Here, we established a rapid method of using high performance liquid chromatography (HPLC) to measure serum oxidized albumin in a rat model. We optimized HPLC conditions for rat oxidized albumin. To validate our method, three-week-old male Sprague-Dawley rats were uninephrectomized and treated normal diet, high salt diet or high salt diet with Tempol, a superoxide dismutase (SOD) mimetic. After 4 weeks of treatment, we analyzed serum oxidized albumin. The main findings are listed as below. (i) Our method of oxidized albumin measurement only takes 16 minutes, with an intra-day and inter-day deviation within 1% and a detection limit concentration of 6.4 mg/ml. (ii) Oxidized albumin levels were significantly higher in the high salt diet group than in the normal salt diet group, and this effect was reversed by Tempol. (iii) Oxidized albumin levels also correlated with urinary protein and 8-isoprostane levels. In conclusion, we have established a simple method for evaluating rat serum oxidized albumin using HPLC. Our method is rapid and has an advantage over conventional methods and may be useful for animal models of oxidative stress.

## Introduction

Oxidative stress has elicited high levels of interest in the field of biology for a long time. It has been reported that oxidative stress plays pivotal roles in a variety of disease conditions as well as in aging^1–5^. To measure oxidative stress levels, many biomarkers has been used, such as 8-isoprostane, malondialdehyde (MDA), nitrotyrosine levels, and serum antioxidant capacity. Each of them has distinct characteristics, but does not necessarily reflect a ubiquitous oxidative stress level. To overcome these problems, we and others have investigated the levels of the oxidized form of albumin as a new marker of oxidative stress^6–9^. Albumin is a mixture of reduced albumin (mercaptalbumin) and oxidized albumin (non-mercaptalbumin) in extracellular fluid such as serum. Reduced albumin has one free sulfhydryl group in Cys-34, while oxidized albumin has a ligand bound to the sulfhydryl group in Cys-34^9^. Reduced albumin is bound with its mixed disulphide with cysteine or glutathione^8^. Oxidized albumin has more oxidized products such as sulfenic (-SOH), sulfinic (-SO_2_H) or sulfonic (-SO_3_H) states^9^.

A former study reported that oxidized rat serum albumin cannot be clearly separated from reduced albumin by conventional HPLC method^8^. Hayashi T *et al*. has developed a method by HPLC which can separate both reduced and oxidize albumin in rat serum^9^. However, Hayashi’s method takes about one hour to measure one sample in room temperature. Here, we established a simple and rapid method for measuring oxidized albumin in rat serum. We validated this method by using an established rat model of high oxidative stress which also demonstrated proteinuria and hypertension^10–12^.

## Results

### Separation of rat oxidized and reduced albumin by using HPLC

**Figure 1:**
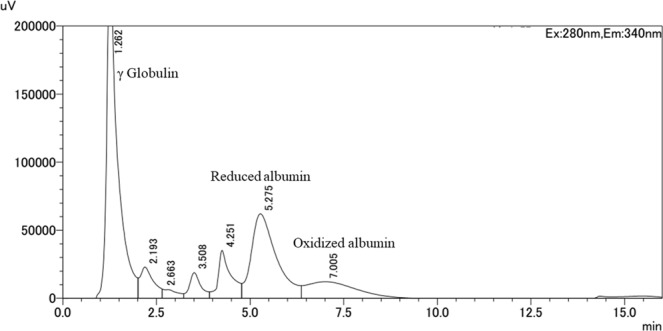
Chromatograph of reduced albumin and oxidized albumin from a healthy rat. Oxidized albumin% was calculated from this formula: Oxidized albumin% = oxidized albumin / (oxidized albumin + reduced albumin) *100%.

By systematically screening of measurement conditions, we eventually determined the optimal condition for measuring rat oxidized and reduced albumin: 25 mM phosphoric acid buffer with 60 mM sodium sulfate, plus 1.5% ethanol (solution I, pH 5.3) and 1000 mM magnesium chloride (solution II). After balancing the column, the flow rate was set to 1 ml/min. The oven temperature was set to 40 °C, and the samples volume was 3 microliters. The whole process of the serum analysis lasted for 12 minutes, and the linear gradient was 0–65% from solution I to solution II. Thus, only a total time of 16 minutes was needed to analyze one sample (including the column balancing and sample analysis times). A representative chromatograph is shown in Fig. 1. Compared with the measurement conditions of human serum albumin, the major differences are ethanol, pH value and linear gradient time. The comparison is summarized in the supplemental Table 2.

### Reproducibility of analysis method

**Figure 2:**
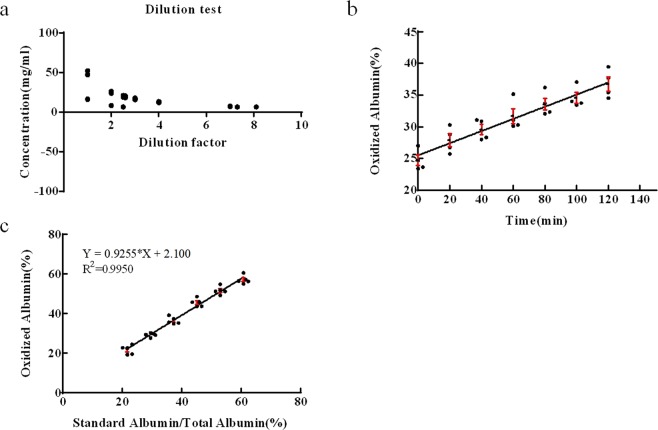
Effects of dilution factor, time changes and standard albumin concentration on oxidized albumin%. (**a**) Dilution test. We diluted serum samples with PBS. The minimal concentration that can be measured by our method is 6.4 mg/ml in serum. (**b**) Time-dependent oxidation of albumin. Samples were left at room temperature before analysis. The horizontal axis indicates the time that the sample was left at room temperature (0 min, 20 min, 40 min, 60 min, 80 min, 100 min, or 120 min). The vertical axis indicates the percentage of oxidized albumin of each sample. The samples used were from healthy rats. (**c**) Standard albumin/total albumin% dependence on oxidation of albumin. Standard albumin was added in 8% increments, and oxidized albumin% was increased linearly.

The reproducibility of our analysis method is summarized in Supplemental Table 1. The CV values of reproducibility for inter-day and intra-day were 0.77% and 0.81%, respectively. We performed multiple dilutions, and the minimal detectable concentration of oxidized albumin was 6.4 mg/ml (Fig. 2a).

Our result showed that 20 minutes of incubation at room temperature caused a significant increase in the percentage of oxidized albumin in a sample, and that the oxidation of albumin is time-dependent (Fig. 2b). We named this process “auto-oxidation”. Serum albumin could be auto-oxidized at room temperature after 20 minutes (Fig. 2b).

We also conducted an “interference evaluation” study. In brief, we used commercially available oxidized albumin standards to calculate the percentage of oxidized albumin, and demonstrated a positive correlation between standard albumin/total albumin and oxidized albumin (Fig. 2c).

### Validation of method in a rat model of proteinuria and hypertension

**Figure 3:**
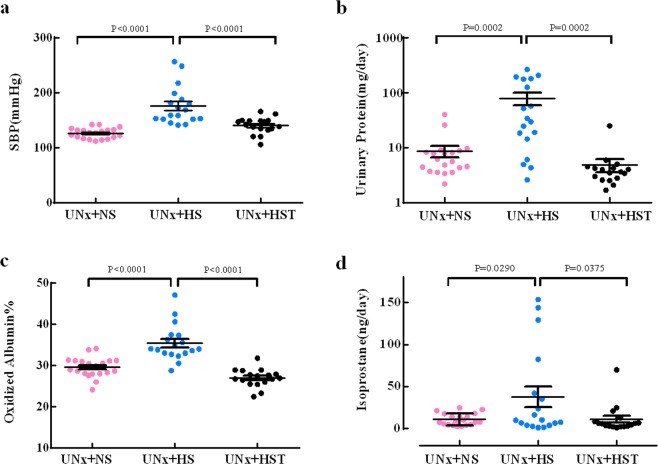
Validation study of oxidized albumin using a rat model of proteinuria and hypertension. (**a**) The systolic blood pressure of the UNx + NS group (n = 20), UNx + HS group (n = 18), and UNx + HST (n = 17) for 4 weeks. The values are shown as the AVE ± SEM. (**b**) The urinary protein level of the proteinuria and hypertension model. The UNx + NS group (n = 20), UNx + HS group (n = 18), and UNx + HST group (n = 17) were evaluated. The values are shown as the AVE ± SEM. (All urinary protein levels were logarithmically transformed prior to statistical analysis by ANOVA with Tukey’s post hoc test because they had a heteroscedastic distribution). (**c**) Oxidized albumin% of the proteinuria and hypertension model. The UNx + NS group (n = 20), UNx + HS group (n = 18), and UNx + HST group (n = 17) were evaluated. The values are shown as the AVE ± SEM. (**d**) The 8-isoprostane level of the proteinuria and hypertension model. The UNx + NS group (n = 20), UNx + HS group (n = 18), and UNx + HST group (n = 17) were evaluated. The values are shown as AVE ± SEM.

We validate our method of measuring oxidized albumin in an established rat model of proteinuria and hypertension^10^. As expected, high salt-loading resulted in significantly higher systolic blood pressure (176.1 ± 34.6 mmHg) in rats with uninephrectomy (UNx) compared to normal salt treated UNx rats (126.4 ± 9.4 mmHg), and additional Tempol in drinking water attenuated high salt loading-induced elevation in systolic blood pressure (140.4 ± 15.0 mmHg) in rats (Fig. 3a).

The urinary protein level in the high salt diet group was significantly higher (78.75 ± 87.13 mg /day) than that from normal salt diet group (8.59 ± 8.95 mg/day), and after treatment with Tempol, the effect of high salt diet on urinary protein was abolished (4.82 ± 5.29 mg /day) (Fig. 3b).

Serum oxidized albumin in high salt diet group (35.43% ± 4.39%) was significantly higher compared to that from normal salt diet group, and Tempol significantly reversed this effect of high salt loading (26.98% ± 2.19%) (Fig. 3c).

Consistent with previous reports^13–15^, the 8-isoprostane level in high salt diet group (36.7 ± 45.9 ng/day) was significantly higher than that in normal salt diet group (10.5 ± 7.4 ng/day), and Tempol reversed this effect (11.0 ± 16.6 ng/day) (Fig. 3d).

**Figure 4:**
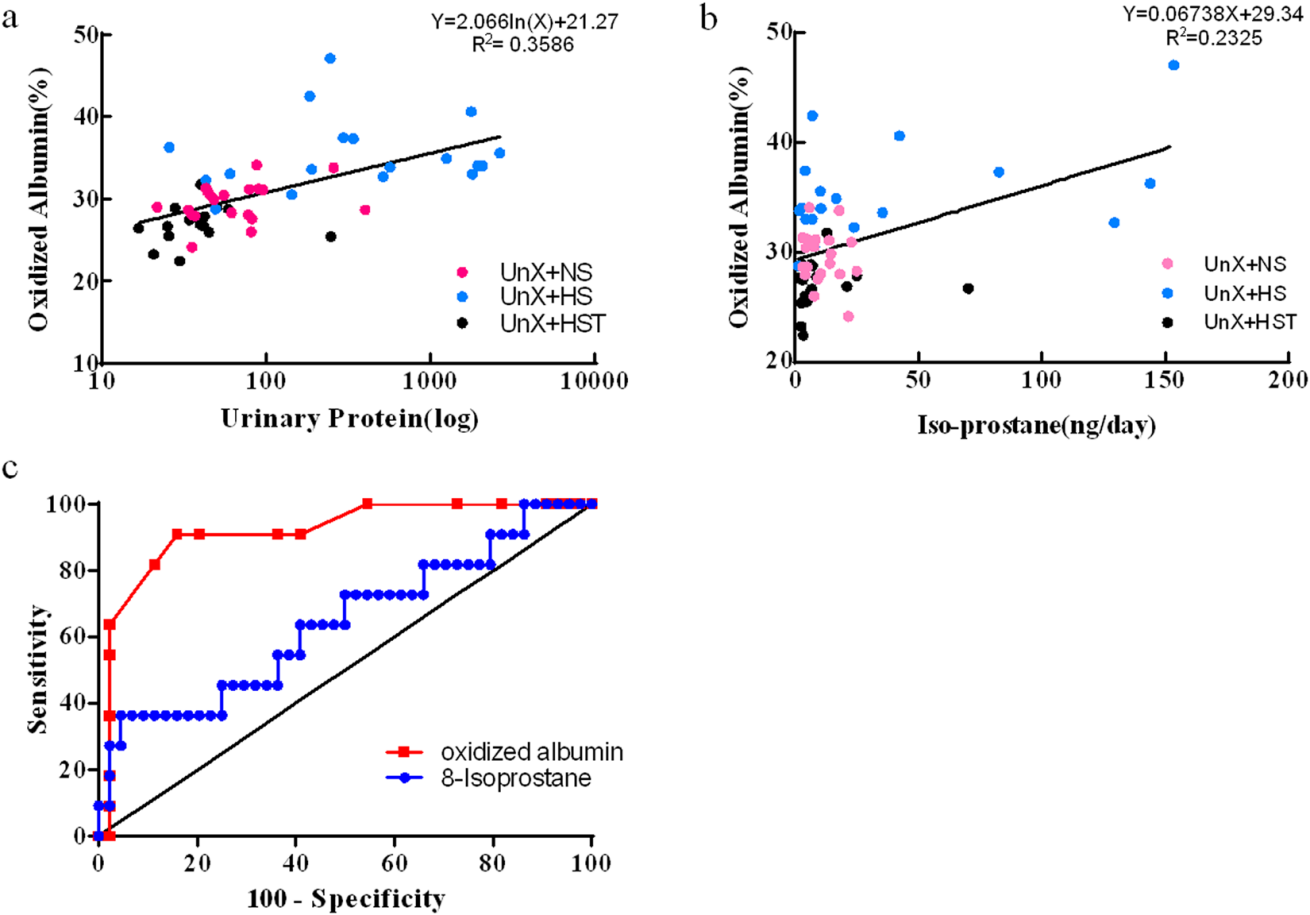
Correlation with conventional method. (**a**) Correlation between oxidized albumin% and urinary protein. The UNx + NS group (n = 20), UNx + HS group (n = 18), and UNx + HST group (n = 17) were evaluated. The values are shown as the AVE ± SEM. (**b**) Correlation between oxidized albumin% and 8-isoprostane.The UNx + NS group (n = 20), UNx + HS group (n = 18), UNx + HST group (n = 17) were evaluated. The values are shown as the AVE ± SEM. (**c**) ROC curves for markers. Receiver operating characteristic (ROC) curves were drawn for oxidized albumin and 8-isoprostane. The area under the curve (AUC) for 8-isoprostane was 0.643, and the AUC curve for oxidized albumin was 0.917, which was an advanced marker compared to 8-isoprostane.

There are positive correlations between oxidized albumin% and both proteinuria (Fig. 4a) and 8-isoprostane (Fig. 4b). Based on the ROC curve, the areas under the curve (AUCs) were 0.643 for urinary 8-isoprostane and 0.917 for oxidized albumin, indicating that oxidized albumin is a new biomarker with greater sensitivity for evaluating oxidative stress, compared to a traditional marker, urinary 8-isoprostane (p < 0.01) (Fig. 4c).

## Discussion

In the present study, we described a simple, adapted method for the measurement of rat oxidized albumin. The total time taken to measure oxidized albumin with our method was only a short 16 minutes, with an intra-day and inter-day deviation within 1% and a detection limit at a concentration of 6.4 mg/ml. Our method is sensitive and rapid, and has an advantage over conventional methods and may be useful for future studies of animal models of oxidative stress.

The gradual increase in oxidized albumin in the intra-day reproducibility analysis may resulted from auto-oxidation. Our experiments have shown that auto-oxidation occurs as quickly as 20 minutes, making it necessary to measure the samples over a short period of time. The total time taken to analyze a sample using our method is as short as 16 minutes (column equilibrating time included). Our method is rapid and capable of separating peaks clearer than former reports^8,9^.

For the peaks to be sharp and be clearly separated, we needed optimal concentrations of magnesium chloride and ethanol. The acidity of the solutions are also important when combining solutions I and II, whereby the acidic II solution was added gradually to solution I to obtain an optimized linear gradient. The pH values of solution I are 6.0 and 5.3 in the human and rat, respectively, which is due to the isoelectric point difference between humans and rats. Both the ion-exchange and hydrophilic interaction of the resin can contribute to the separation of oxidized albumin and reduced albumin by the column, which is further enhanced by varying magnesium concentration and the pH of solution I. It is also noteworthy that there are also limitations of our method. It is reported that both in human and rat cases, oxidized albumin is the mixture of NA-1(Non-mercaptalbumin-1) and NA-2(Non-mercaptalbumin-2)^16–18^.These two states of oxidized albumin may represent different pathophysiology^19^. In our model, the new method has not shown separation of these two peaks. Further research in different model should be conducted.

For the interference study, we did an “interference evaluation”. As standard oxidized albumin (100% oxidized) increased by 8%, the percentage of oxidized albumin increased by 7.42% (AVE). There is a positive correlation between the percentage of standard albumin/total albumin and oxidation albumin. Our result showed that our method for measuring oxidized albumin closely reflect the actual amount of standard albumin. Based on the intra-day and inter-day reproducibility, the method was sensitive enough to measure rat oxidized albumin at a concentration as low as 6.4 mg/ml.

Previous reports have shown that many diseases are associated with high oxidative stress levels in both humans^6,20,21^ and animal models^22–26^. *In vitro* studies have also shown that oxidized albumin itself is toxic^27^. Previous animal study has also shown oxidative changes in the blood and serum albumin in rats with monoarthritis and polyarthritis^28^. To validate our method, we utilized an established rat model of high in oxidative stress which is associated with proteinuria and hypertension^10^. The results from our study showed that the percentage of oxidized albumin was significantly higher in the disease model than in the control group and that this change was reversed by the anti-oxidant drug Tempol. These results were consistent with human renal function data^7,29^. To further confirm our results, we have compared serum oxidized albumin with urinary 8-isoprostane levels, which is a commonly used marker to indicate the oxidative stress. The high salt group also showed a significantly higher 8-isoprostane level than the other two groups, and it positively correlated with our measurement of serum oxidized albumin. These findings suggest that our method of measuring oxidized albumin is in accordance with the currently using marker 8-isoprostane and has the potential to be applied to other disease models. Our correlation data between oxidized albumin and urinary protein also suggests that measured oxidized albumin may be a better marker for oxidative stress-related organ damage.

8-Isoprostane is formed by the peroxidation of arachidonic acid^30^, which is found in cell membrane phospholipids and is a target of reactive oxygen species^31^, whilst urinary protein is a representative marker to evaluate renal damage^10,32^ and reflects glomerular damage. A previous report shows that 8-isoprostane is not a prerequisite for renal damage^33^. Taken together, oxidized albumin is a useful marker for oxidative stress-induced kidney disease, and appear to be an advanced marker compared to 8-isoprostane. A reduction in the percentage of oxidized albumin may also be a target for the prevention of renal diseases.

In conclusion, we have established a simple and rapid method for measuring oxidative stress levels with oxidized albumin. We validated this method by using an established rat model of proteinuria and hypertension which demonstrate high levels of oxidized albumin and 8-isoprostane. Our method is sensitive and rapid, and has an advantage over conventional methods and may be useful for future studies of animal models of oxidative stress.

## Materials and Methods

### Animals

Three-week-old male Sprague Dawley (SD) rats (body weights ranging from 40 g to 50 g) were subjected to left uninephrectomy and then randomly divided into the following 3 groups: the normal salt diet (UNx + NS, 0.3% NaCl, n = 20); high salt diet (UNx + HS, 8% NaCl, n = 18) and high salt diet treated with a superoxide-dismutase mimetic, namely, 4-hydroxy-2,2,6,6-tetramethyl-piperidine-N-oxyl, also known as Tempol (UNx + HST, 8% NaCl, 1 mmol/kg/day in drinking water, n = 17). After 4 weeks of treatment, blood samples were collected by cardiac puncture.

Rats were maintained in a humidity- (60 ± 5%), temperature- (23 ± 1.5 °C), and light cycle-regulated (0700–1900 h) room and had free access to food and drinking water. Reasearch Ethics Committee of the University of Tokyo approved this investigation (No:P14–145), which was conducted according to the guidelines for the care and use of laboratory animals of the University of Tokyo, Graduate School of Medicine.

Body weight and systolic blood pressure (SBP) were measured prior to sacrifice. SBP was measured using the tail-cuff method (P-98A; Softron, Tokyo, Japan). Twenty-four-hour urine samples were collected at the end of the 4-week treatment using metabolic cages to measure both the urinary protein and the 8-isoprostane levels.

### Condition establishment for HPLC

The HPLC system (LabSolutions system; Shimazu Co., Ltd, Kyoto, Japan) comprised of a degasser (DGU20A3R), two pumps (LC-20AT), an autosampler (SIL30AC), a thermostatic oven (CTO-20AC), a fluorescence detector (RF-20Axs) and a system controller (CBM-20 A).

The polyvinyl alcohol cross-linked gel (9 µm in diameter) (Asahipak GS-520; AsahiKasei Co., Ltd, Tokyo, Japan) which was dried in a vacuum for more than 16 hours was then suspended in 10 ml of dimethyl sulfoxide (DMSO; Tokyo Chemical Industry Co., Ltd, Tokyo, Japan) per gram of the dried gel. Next, to suspend the gel, 20 mmol of epichlorohydrin were added to per gram of the dried gel for 20 hours at 30 °C. The activated gel was then filtrated, followed by an addition of 10% aqueous solution of diethyl amine (Wako Pure Chemical Industries, Ltd, Osaka, Japan) for another 20 hours. Finally, the gel was packed in a stainless column (50 × 7.6 mm I.D.). By using the above prepared column which is of high resolution and anion exchanged, the analysis condition to separate rat reduced albumin and oxidized albumin was screened by optimizing the factors—buffers, pH value, ethanol concentration, flow rate, the linear gradient of magnesium concentration, oven temperature and sample volume.

All of the reagents were either HPLC grade or a special grade (Wako Pure Chemical Industries, Ltd, Osaka, Japan).

The fluorescence detection wavelengths were 280 nm and 340 nm. Two eluent buffers contained 60 mM sodium sulfate and 25 mM phosphoric acid buffer. Three microliters of serum or plasma samples was directly injected into the system. The flow rate was 1 ml/min, and the analytical temperature was 40 °C. The peaks for reduced albumin and oxidized albumin were then confirmed. Based on the peak areas, the results were expressed as Oxidized albumin% = Oxidized albumin/(Reduced albumin + Oxidized albumin) × 100%^7,34,35^.

### Evaluation of method performance

The intra-day reproducibility was checked 10 times continuously in one day, and inter-day reproducibility was checked on 10 continuous days.

### Sensitivity of the analysis method

To study the detection limit of rat oxidized albumin in our method, we used serum samples from healthy rats and diluted them with PBS. Oxidized albumin level was measured as described above.

### Evaluation of auto-oxidation

To investigate the time-dependent auto-oxidation of albumin at room temperature, we measured control samples of serum albumin every 20 minutes for a total 120 minutes.

### Interference study

We added standard albumin (100% oxidized albumin, Supplemental Fig. 1) to the rat serum samples following a linear concentration gradient from 0% to 48% (the sample concentration gradient increased by 8%) and then measured oxidized albumin level using HPLC.

### Method for evaluation of the 8-isoprostane concentration

Urine samples of the rats were centrifuged at 10,000 rpm for 5 minutes. The urine samples from rats fed normal salt diets were diluted 5 times, whilst samples from rats fed a high salt diet or high salt diet plus Tempol were not diluted.

According to the manufacturer’s instructions, we measured 8-isoprostane levels with the 8-iso-PGF2α Elisa kit (Enzo Life Sciences, Inc. Farmingdale, New York, USA). Briefly, the plate was incubated with the sample at room temperature on a plate shaker at 120 rpm for 120 minutes and then washed 5 times using washing solution. After incubating at room temperature with a solution of p-nitrophenyl phosphate in buffer for 45 minutes without shaking and adding stop solution, the optical density was measured at 420 nm. The concentration of 8-isoprostane was calculated. Urine samples and standard samples were all run in duplicate.

### Statistical analysis

Data are expressed as the mean ± SEM. All urinary protein levels were logarithmically transformed prior to statistical analysis by ANOVA with Tukey’s post hoc test because they had a heteroscedastic distribution. Statistical analysis among the 3 groups was performed by one-way ANOVA followed by Tukey’s test. The receiver operating characteristic (ROC) curve for oxidized albumin and 8-isoprostane was drawn to compare the diagnostic value. P < 0.05 was considered to be statistically significant.

## Supplementary information

Supplemental information

## Data Availability

The authors confirm that the data supporting the findings of this study are available within the article and its supplementary materials.
