# Supplementary material for: A rapid method for measuring serum oxidized albumin in a rat model of proteinuria and hypertension
Source: Sci Rep. 2019 Jun 13;9:8620. doi: 10.1038/s41598-019-45134-x (PMC6565692; doi:10.1038/s41598-019-45134-x)
Supplement: Supplementary file 1 — Supplemental information [file 41598_2019_45134_MOESM1_ESM.docx]

Supplemental Materials

**A rapid method for measuring serum oxidized albumin in a rat model of proteinuria and hypertension**

Authors: Beibei Liu^1,2^, Keiko Yasukawa^1^, Suang Suang Koid^1,2^, Alimila Yeerbolati^1,2^, Latapati Reheman^1,2^, Conghui Wang^1,2^, Yutaka Yatomi^1^, Tatsuo Shimosawa^2 *^

Affiliation: ^1^ Department of Clinical Laboratory, The University of Tokyo Hospital, Tokyo, Japan

^2^ Department of Clinical Laboratory, International University of Health and Welfare School of Medicine, Chiba, Japan

Correspondence: Dr. Tatsuo Shimosawa

Email: [tshimo-tky@umin.ac.jp](mailto:tshimo-tky@umin.ac.jp)

Supplemental Figure. 1 The representative chromatograph of the standard albumin (100% purity) which we used for the interference study (Fig 2c)

by means of HPLC.


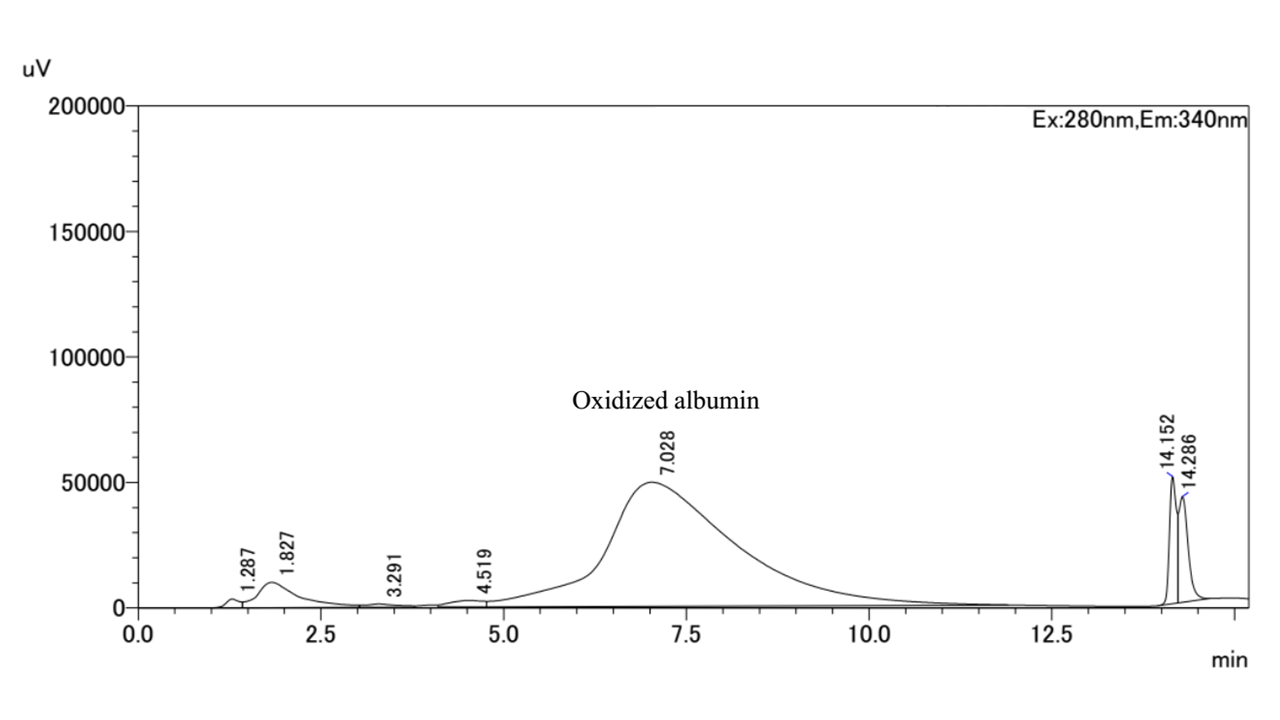


Supplemental Table. 1 Inter-day and Intra-day reproducibility (%) for analysis conditions

|  | 1 | 2 | 3 | 4 | 5 | 6 | 7 | 8 | 9 | 10 | AVE. | SD. | CV. |
| --- | --- | --- | --- | --- | --- | --- | --- | --- | --- | --- | --- | --- | --- |
| Inter-day Reproducibility (%) | 71.25 | 70.52 | 71.96 | 71.49 | 70.15 | 70.52 | 71.31 | 71.68 | 70.88 | 70.80 | 71.06 | 0.55 | 0.77 |
| Intra-day Reproducibility (%) | 72.83 | 72.46 | 72.36 | 71.90 | 71.79 | 71.79 | 71.43 | 71.48 | 71.16 | 70.83 | 71.80 | 0.58 | 0.81 |

Supplemental Table. 2 Human and rat measurement condition comparison.

| Measurement conditions | Rat | Human[1] |
| --- | --- | --- |
| Buffer | 25mM phosphoric buffer with 60mM sodium sulfate.  High conc. Magnesium chloride | 25mM phosphoric buffer with 60mM sodium sulfate.  High conc. Magnesium chloride |
| Flow rate | 1m/min | 1m/min |
| Oven temperature | 40°C | 40°C |
| Sample volume | 3μl | 3μl |
| Ethanol | 1.5% | No use of Ethanol |
| pH Value | pH 5.3 | pH 6.0 |
| Linear gradient time | 12min | 7.5min |

*[1] Yasukawa, K., Shimosawa, T., Okubo, S. & Yatomi, Y. A simple, rapid, and validated high-performance liquid chromatography method suitable for clinical measurements of human mercaptalbumin and nonmercaptalbumin. Ann. Clin. Biochem. 55, 121–127 (2017).*
